# Effect of Partial Substitution of Zr for Ti Solvent on Young’s Modulus, Strength, and Biocompatibility in Beta Ti Alloy

**DOI:** 10.3390/ma17112548

**Published:** 2024-05-25

**Authors:** Yusuke Nomura, Mio Okada, Tomoyo Manaka, Taiki Tsuchiya, Mami Iwasaki, Kenji Matsuda, Takuya Ishimoto

**Affiliations:** 1 Department of Materials Design and Engineering, Faculty of Sustainable Design, University of Toyama, Toyama 930-8555, Japan; m23c1618@ems.u-toyama.ac.jp (Y.N.); sea.sound.nba@gmail.com (M.O.); tsuchiya@sus.u-toyama.ac.jp (T.T.); matsuda@sus.u-toyama.ac.jp (K.M.); 2 Aluminium Research Center, University of Toyama, Toyama 930-8555, Japan; manaka@sus.u-toyama.ac.jp; 3 Department of Mechanical Engineering, University of Toyama, Toyama 930-8555, Japan; miwasaki@eng.u-toyama.ac.jp

**Keywords:** low Young’s modulus, high strength, solid solution strengthening, β-Ti, mechanical osteocompatibility

## Abstract

In orthopedics and dentistry, there is an urgent need to obtain low-stiffness implants that suppress the stress shielding caused by the use of metallic implants. In this study, we aimed to fabricate alloys that can reduce the stiffness by increasing the strength while maintaining a low Young’s modulus based on the metastable β-Ti alloy. We designed alloys in which Ti was partially replaced by Zr based on the ISO-approved metastable β-Ti alloy Ti-15Mo-5Zr-3Al. All alloys prepared by arc melting and subsequent solution treatment showed a single β-phase solid solution, with no formation of the ω-phase. The alloys exhibited a low Young’s modulus equivalent to that of Ti-15Mo-5Zr-3Al and a high strength superior to that of Ti-15Mo-5Zr-3Al and Ti-6Al-4V. This strengthening was presumed to be due to solid-solution strengthening. The biocompatibility of the alloys was as good as or better than that of Ti-6Al-4V. These alloys have potential as metallic materials suitable for biomedical applications.

## 1. Introduction

The stress shielding of bones associated with the application of metallic implants has been one of the major challenges in orthopedics and dentistry for decades. Stress shielding refers to the phenomenon in which the bone is not subjected to healthy stress (or strain) owing to the high stiffness of metallic implants. Reducing the Young’s modulus is a promising strategy to reduce implant stiffness and inhibit stress shielding [1]. The Young’s modulus of the Ti-6Al-4V (mass%) alloy, currently the most commonly used material for implants, is 110 GPa, which is several times greater than the modulus of bone (20–40 GPa).

One way to reduce the Young’s modulus (apparent Young’s modulus) is to make the material porous [2]. However, porous materials cause significant degradation of their fatigue properties [3], limiting their use as implants. Porous materials have been clinically used for the outer surface of acetabular cups and the internal structure of spinal cages [4], but not in areas subjected to severe cyclic loading, such as artificial hip joints or bone plates for temporary fixation of fractures.

Metastable β-Ti alloys with low Young’s moduli and bcc structures have been extensively studied and developed [1]. There are several guiding principles for the development of Ti alloys with low Young’s moduli, one of which is the reduction of the electron-to-atom ratio (*e*/*a*) [5]. The lowness of the *e*/*a* is an indicator of the instability of the β phase; it has been shown that a reduction in the *e*/*a* reduces the Young’s modulus of polycrystalline materials [6], as estimated by the Hill approximation [7]. The modulus decreased monotonically with a reduction in the *e*/*a* [6]. The *e/a* ratio was manipulated by adding appropriate amounts of the β- and α-phase stabilizers to the Ti solvent in a balanced manner.

**Figure 1:**
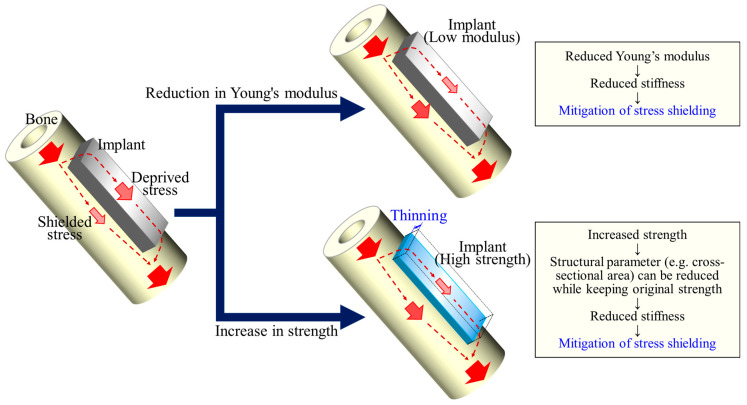
A schematic illustration showing that a higher strength of the implant material contributes to a reduction in the stiffness of the implant as well as a reduction in the Young’s modulus, leading to a reduction in stress shielding to the bone.

Another possible strategy for reducing the stiffness of an implant is to increase its strength [8]. Assuming uniaxial loading for simplicity, if the strength is increased x times (x > 1), the amount of material or cross-sectional area required to withstand the same load can be reduced by 1/x. When the Young’s modulus of the material remains unchanged, this reduction in the cross-sectional area results in a proportional reduction in the stiffness. In other words, the higher strength of the implant material, as well as the reduction in Young’s modulus, contributes to a reduction in the stiffness of the implant, leading to a reduction in stress shielding to the bone (Figure 1).

In this study, we attempted an alloy design to increase the strength of β-Ti alloys while maintaining their intrinsically low Young’s modulus. As a reference metastable β-Ti alloy, we used Ti-15Mo-5Zr-3Al (mass%) alloy, which is an ISO-approved (ISO 5832-14 [9]) β-Ti alloy with an *e*/*a* as low as 4.10 and a polycrystalline Young’s modulus of 80–90 GPa [10]. Although there are generally several selections for strengthening metals, this study focuses on solid solution strengthening. The objective of this study is to enhance solid solution strengthening by adding elements with different atomic sizes while maintaining an *e*/*a* of 4.10 for a low Young’s modulus. Specifically, in this study, the Ti in the Ti-15Mo-5Zr-3Al alloy was partially replaced by Zr, whose valence electron number is equal to that of Ti (4), for strengthening. The mechanical properties and biocompatibility of the designed alloys are reported.

## 2. Materials and Methods

### 2.1. Alloy Composition Design and Preparation of Ingot

The alloy compositions were determined based on the Ti-15Mo-5Zr-3Al (Ti_83.3_Mo_8.1_Zr_2.8_Al_5.8_ in at%), with the *e*/*a* unchanged; that is, the molar concentrations of Mo and Al were fixed, and the Ti in the solvent of the alloy was partially replaced by Zr in a stepwise manner. The atomic radii and valence electron numbers of the elements used in this study are presented in Table 1. The compositions and *e*/*a* values of the alloys are summarized in Table 2. The alloys are denoted as Zr0, Zr25, Zr50, and Zr75 based on the concentration ratio of Ti and Zr. For Zr25, for example, the Ti/Zr concentration ratio was 3:1. The *e*/*a* was 4.10 for all alloys, which is the same as for Ti-15Mo-5Zr-3Al. Ti-15Mo-5Zr-3Al and Ti-6Al-4V were also prepared for comparison purposes.

The alloy ingots were prepared using high-purity metals of Ti (>99.9%), Zr (>99.6%), Mo (>99.9%), Al (>99.99%), and V (>99.7%) by arc melting in a high-purity argon atmosphere. The ingots were inverted and remelted at least 15 times and maintained in a liquid state for 120 s during each melting event to ensure the homogeneity of the chemical composition. Using thermodynamic software (Thermo-Calc 2022a, Thermo-Calc Software, Solna, Sweden) and a database of Ti (TCTI4, Thermo-Calc Software), we estimated the temperature range for β single-phase for each alloy. The ingots were solution-treated in the β single-phase region at 1000 °C for 3.6 ks in vacuum, after which they were quenched with ice water.

### 2.2. Microstructural Observation

The microstructures were observed using optical microscopy (OM; BX53M, Olympus, Tokyo, Japan). The specimens for OM were cut using electrical discharge machining, mechanically polished using SiC waterproof papers up to #4000 grit, and mirror-polished with a colloidal silica suspension. The surfaces of the specimens were then etched with a solution consisting of 2% hydrofluoric acid, 12% nitric acid, and 86% water at 0 °C.

### 2.3. Phase Identification by X-ray Diffraction

The phase compositions of the alloys were identified by X-ray diffraction (XRD; Ultima IV, Rigaku, Tokyo, Japan) with Cu-Kα radiation at an accelerating voltage and currents of 40 kV and 40 mA, respectively. The measurements were performed using a 2*θ* scan from 30° to 90° at a scan rate of 1.0°/min and a scan interval of 0.02°.

### 2.4. Mechanical Property Evaluation by Compression Testing

The Young’s modulus (*E*) and yield stress (0.2% proof stress: *σ*_0.2_) were analyzed using a compression test. Rectangular specimens (3 mm × 3 mm × 7.5 mm) were cut from the ingot using electrical discharge machining, and the 7.5 mm side was parallel to the compressive force. The compression tests (*n* = 3) were conducted using an Instron-type testing machine (AGX-V, Shimadzu, Kyoto, Japan) at an initial strain rate of 0.01 /min at room temperature. Strain gauges (KFGS-2N-120-C1-11 L1M2R, Kyowa Electronic Instruments, Tokyo, Japan) were attached to the specimen sides to measure the strain required to determine the Young’s modulus. Mechanical osteocompatibility was defined as the yield stress divided by the Young’s modulus (*σ*_0.2_/*E*). The mechanical osteocompatibility was calculated for each specimen.

### 2.5. Microstructural Analysis by Transmission Electron Microscopy

Microstructural analysis was performed using transmission electron microscopy (TEM; EM-002B, Topcon, Tokyo, Japan), with an acceleration voltage of 120 kV. The TEM specimens were prepared using a focused ion beam system (FIB; FB-2100, Hitachi High-Teck, Tokyo, Japan).

### 2.6. Evaluation of Biocompatibility

Five specimens with dimensions of 5 mm × 5 mm × 1 mm were cut from the ingot using electrical discharge machining and polished with #1000, #1200, and #2400 emery paper. The surfaces were mirror-polished using colloidal silica. After polishing, the specimens were ultrasonically cleaned in acetone and ethanol and then rinsed in filtered Milli-Q water. They were then autoclaved at 121 °C for 30 min before cell culture studies.

Normal mouse calvarial preosteoblast cells (MC3T3-E1, Riken Cell Bank, Tsukuba, Japan) were cultured in a modified Eagle’s medium (α-MEM) (Fujifilm Wako, Osaka, Japan) supplemented with 10% fetal bovine rerum (FBS, Corning, New York, NY, USA) and 1% penicillin and streptomycin (Fujifilm Wako), and incubated at 37 °C in a fully humidified atmosphere (95% air, 5% CO_2_). The medium was exchanged every two days, and the cells were passaged by trypsinization when they reached 80% confluency. MC3T3-E1 cells were seeded on the specimens placed in the wells of 48-well plates (Corning, New York, NY, USA) in 250 µL of medium at a density of 6 × 10^4^ cells/mL. After 12 and 24 h of culture, the cells cultured on the specimen surface were stained with May–Grünwald–Giemsa (MGG).

The specimens were washed twice with phosphate-buffered saline (PBS), and stained with May–Grünwald solution (Muto Pure Chemicals, Tokyo, Japan) for 2 min. An equal volume of Giemsa solution (Muto Pure Chemicals) diluted 1:20 in phosphate buffer was then mixed and stained for an additional 15 min. Subsequently, the specimens were washed twice with filtered Milli-Q water and dried. The specimens were observed under an optical microscope, and the cell numbers were counted.

### 2.7. Statistical Analyses

The quantitative results are expressed as the means ± standard deviations. For the comparison of the data between the cell density for the 12 and 24 h cultures, a two-tailed *t*-test was used. To compare the data among the groups, one-way analysis of variance and post hoc Tukey’s HSD multiple comparisons were performed. Statistical significance was set at *p* < 0.05. SPSS version 25 software (IBM, New York, USA) for Microsoft Windows was used for the statistical analyses.

## 3. Results and Discussion

### 3.1. Phase Constitution and Microstructure

**Figure 2:**
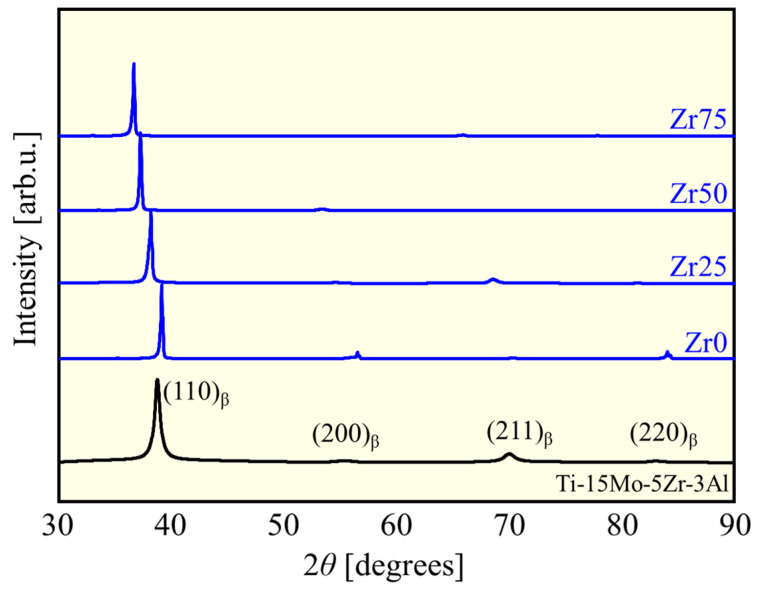
XRD patterns of the designed alloys and Ti-15Mo-5Zr-3Al.

The XRD profiles of the fabricated alloys and Ti-15Mo-5Zr-3Al are shown in Figure 2. In all XRD profiles, peaks derived from the bcc phase can be observed, but those of other phases were not identified, indicating that the alloys produced were almost entirely composed of a bcc (β) single phase. The peaks of the β-phase shifted to the lower 2*θ* region with an increase in the concentration of Zr with a larger atomic radius. This corresponds to an increase in the lattice constant, 3.25 Å for Zr0 to 3.46 Å for Zr75, in accordance with Begard’s law, an empirical law in substitutional solid solution, supporting the formation of solid solution [11,12,13,14].

The formation ability of a solid solution in a multicomponent alloy system is often discussed in terms of mixing enthalpy ${∆H}_{mix}$, and the atom size difference parameter δ is defined as follows:

(1) $$∆H_{mix}=4\sum_{i=1,j=1}^{n}x_{i}x_{j}∆H_{i-j}^{mix}\cdot (i\;\neq \;j),$$

(2) $$\delta =\sqrt{\sum_{i=1}^{n}x_{i}\cdot {\left(1-\frac{r_{i}}{r_{ave}}\right)}^{2}},$$

where *x*_i_ is the mole fraction of element *i*, $∆H_{i-j}^{mix}$ is the enthalpy of mixing for binary alloys calculated by Miedema’s model [13], *r*_i_ is the atomic radius of element *i* (Table 1), and *r*_ave_ is the average atomic radius, defined as

(3) $$r_{ave}=\sum_{j=1}^{n}x_{j}r_{j}.$$

The ${∆H}_{mix}$ and *δ* values of each alloy are listed in Table 3. 

The ∆*H*_mix_ became negatively larger with an increasing Zr concentration. This is because $∆H_{Zr-Mo}^{mix}$ (= −6 kJ/mol) and $∆H_{Zr-Al}^{mix}$ (= −44 kJ/mol) are negatively larger than $∆H_{Ti-Mo}^{mix}$ (= −4 kJ/mol) and $∆H_{Ti-Al}^{mix}$ (= −30 kJ/mol) [15]. The δ also tends to be larger for alloys with higher Zr concentrations because the atomic radius differences between Zr and Mo and Zr and Al are larger than those between Ti and Mo and Ti and Al (Table 1). The range of these values falls within the empirical range for obtaining a single-phase solid solution [16]; solid solutions were obtained in this study.

**Figure 3:**
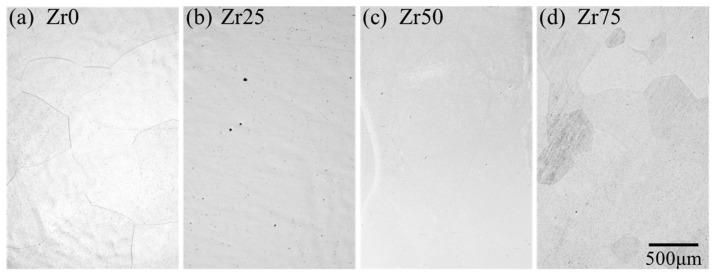
Optical micrographs of the microstructures of the designed alloys.

The typical microstructures of each designed alloy after the solution treatment are shown in Figure 3. All the alloys consist of equiaxed grains with an average grain size of 300–500 μm without precipitates.

### 3.2. Mechanical Properties

**Figure 4:**
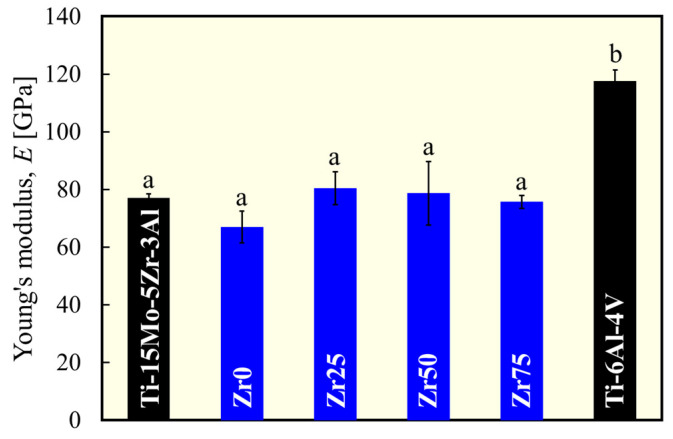
Variation in Young’s modulus of each alloy. a and b indicate statistically homogeneous subgroups, with *p* > 0.05 (Tukey’s HSD multiple comparison test).

**Figure 5:**
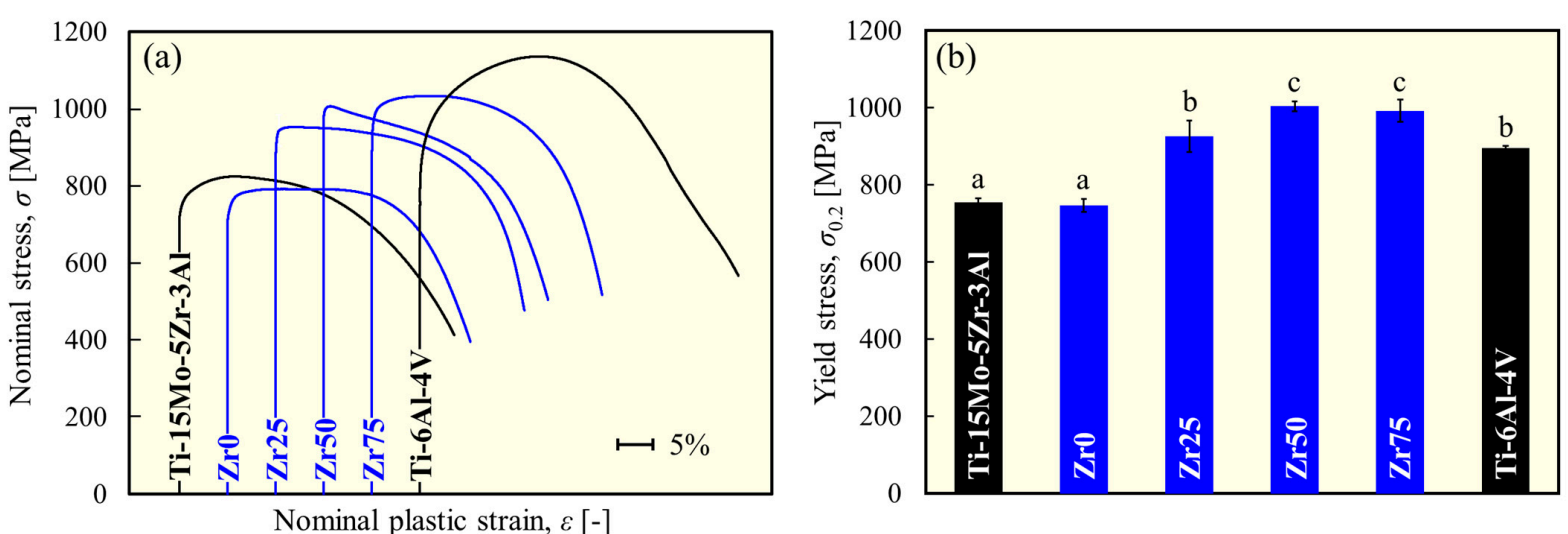
(**a**) Typical stress–plastic strain curve and (**b**) variation in yield stress of each alloy obtained from the compression test. a, b, and c indicate statistically homogeneous subgroups, with *p* > 0.05 (Tukey’s HSD multiple comparison test).

Figure 4 shows the variations in the Young’s modulus for each alloy. Figure 5 depicts the typical stress–plastic strain curve and yield stress. The Young’s modulus and yield stress of Ti-15Mo-5Zr-3Al [17] and Ti-6Al-4V [18] are comparable to the values reported in the literature. The Young’s modulus of each designed alloy is comparable to that of Ti-15Mo-5Zr-3Al but significantly lower than that of Ti-6Al-4V (Figure 4). There was no statistically significant variation in Young’s modulus with the Zr content. This justifies the strategy of this study to maintain a low Young’s modulus by maintaining the *e*/*a* at a low value (4.10), thereby preserving the instability of the β phase. The Young’s modulus values obtained in this study are consistent with the *e*/*a* dependence of the polycrystalline Young’s modulus of β-Ti alloys reported by Tane et al. [6], that is, approximately 70 GPa at *e*/*a* = 4.10. The yield stress of the designed alloys is significantly higher than that of Ti-15Mo-5Zr-3Al, except for Zr0, to which Zr was not added (Figure 5b). Zr50 and Zr75 exhibited the highest yield stresses in this study; almost 1000 MPa was reached, which is more than 30% greater than Ti-15Mo-5Zr-3Al. 

**Figure 6:**
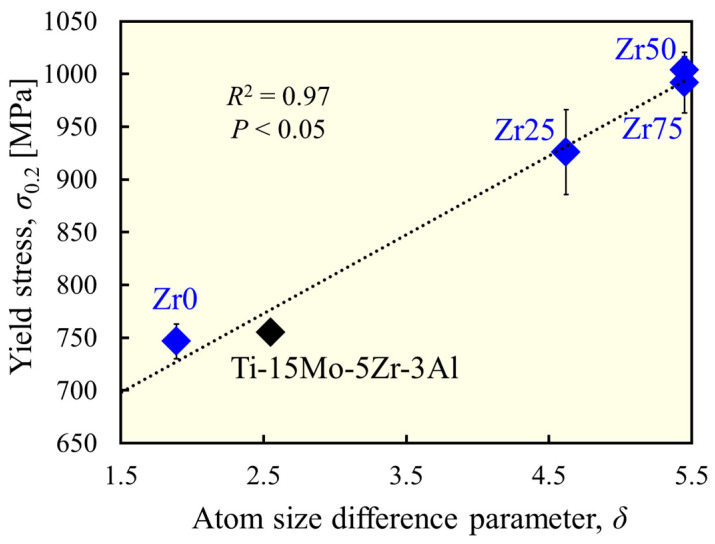
Correlation between atom size difference and yield stress.

In the designed alloys, there were no clear precipitates, and furthermore, the crystal grains were coarse because the present study did not expect grain-boundary strengthening effects. Therefore, the variation in the yield stress in this series of alloys is attributed to the difference in the degree of solid solution strengthening by the addition of Zr. Lattice strains leading to solid solution strengthening were estimated using the δ parameter. Figure 6 shows the correlation between the δ and the yield stress for β-phase alloys. A significant positive correlation (*R*^2^ = 0.97) was found, indicating that the moderate lattice distortion caused by the moderate atomic size difference resulted in a higher strength owing to the solid solution formation and activation of the solid solution strengthening effect.

**Figure 7:**
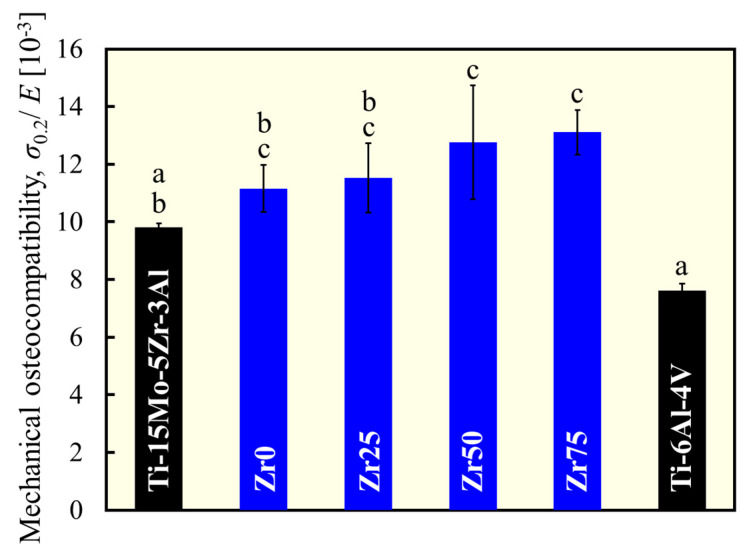
Variation in mechanical osteocompatibility. a, b, and c indicate statistically homogeneous subgroups, with *p* > 0.05 (Tukey’s HSD multiple comparison test).

Mechanical osteocompatibility was defined as the yield stress divided by the Young’s modulus. In this context, the higher the strength and the lower the Young’s modulus, the better the mechanical osteocompatibility. The mechanical osteocompatibility parameters of each alloy are shown in Figure 7. The mechanical osteocompatibilities of Zr0 and Zr25 were significantly greater than those of Ti-6Al-4Al, and those of Zr50 and Zr75 were significantly greater than those of both Ti-6Al-4Al and Ti-15Mo-5Zr-3Al. Therefore, the alloy design concept used in this study works well to improve mechanical osteocompatibility.

### 3.3. ω Phase Formation

**Figure 8:**
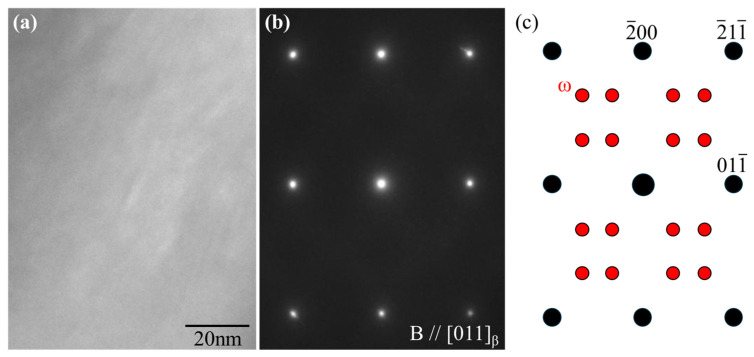
(**a**) Bright-field image and (**b**) electron diffraction patterns of Zr50 alloy obtained along the [110]β zone. (**c**) Key diagram of the electron diffraction spots derived from the β (black) and ω phases (red).

In metastable β-type Ti alloys, a hexagonal ω phase can be formed through ω transformations [19]. The ω phase should be avoided in bone biomaterials because it contributes to increased strength but also increases the Young’s modulus and makes the alloy brittle [20]. Figure 8a,b show the TEM bright-field image and electron diffraction pattern of Zr50. If the ω phase is present in the β matrix, additional diffraction spots would appear between the spots of β phase, as shown in Figure 8c [21,22]. However, the electron diffraction pattern confirms that the ω phase was not formed. In Ti-15Mo-5Zr-3Al, the addition of Al is responsible for the suppression of ω phase formation [23,24]. The suppression effect of Al was maintained in the alloys with increasing Zr concentrations.

### 3.4. Biocompatibility

**Figure 9:**
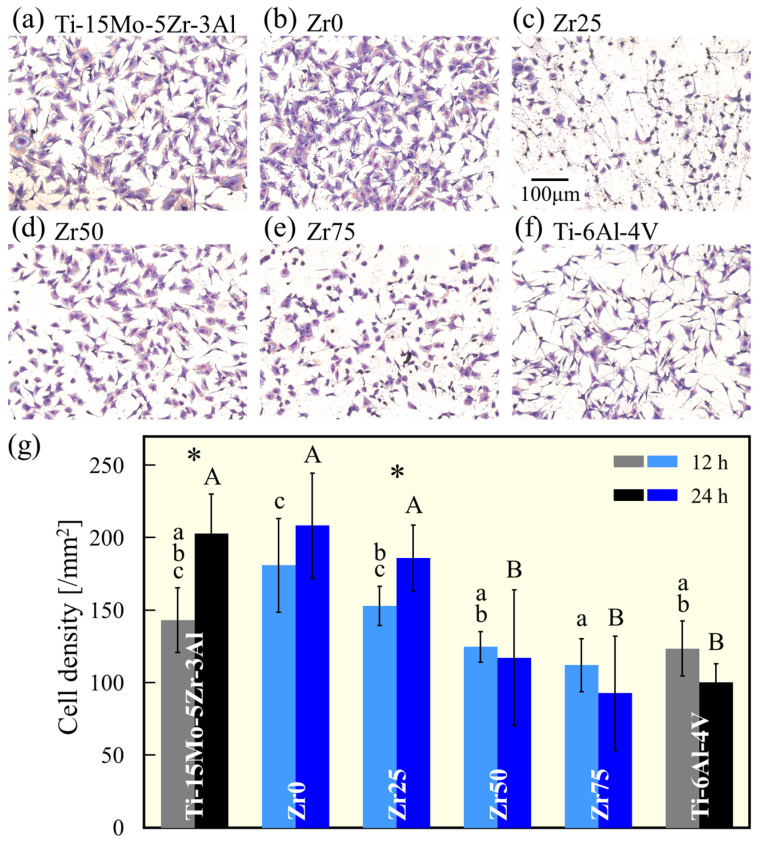
MGG-stained images of MC3T3-E1 osteoblast cells cultured on (**a**) Ti-15Mo-5Zr-3A, (**b**) Zr0, (**c**) Zr25, (**d**) Zr50, (**e**) Zr75, and (**f**) Ti-6Al-4V for 12 h. (**g**) The density of the MC3T3-E1 cells cultured on the alloy specimens for 12 and 24 h. a, b, c, A, and B indicate homogeneous subgroups (*p* > 0.05, Tukey’s HSD multiple comparison test). * Significant difference (*p <* 0.05, *t*-test).

Figure 9a–f show the morphology of MC3T3-E1 cells cultured on the specimens for 12 h. The density of cells adhering to the surface of the sample 12 and 24 h after cell seeding is shown in Figure 9g. The cells were well elongated in all samples. The cell density was similar to that of Ti-15Mo-5Zr-3Al for Zr0 and Zr25 but decreased with an increasing Zr concentration. Similar to the results of this study, the addition of Zr has been reported to reduce the number of adherent cells [25]; however, the effect of Zr addition on cell density and cell viability remains inconsistent in the literature [26]. The cell behavior is largely dependent on the morphological, chemical (e.g., surface oxide composition and/or ion release), and physical (e.g., hardness) states of the surface [26]. The effects of these surface properties, which depend on the addition of Zr, and the long-term biocompatibility of the alloy should be investigated in future studies. However, the value of the findings of this study is that even at Zr75, with the highest Zr concentration of 64.61 at%, the cell density is shown to be comparable to that of Ti-6Al-4V, the most frequently used metallic biomaterial. 

### 3.5. Advantages and Challenges of This Alloy as a Bone Biomaterial

In this study, we obtained alloys with identical or lower Young’s moduli and higher strengths than the currently approved biomaterials for bone implants, Ti-15Mo-5Zr-3Al and Ti-6Al-4V. Many studies have attempted to reduce the Young’s modulus to suppress stress shielding [27]. On the other hand, the degree of stress shielding, or the degree to which stress is removed from the bone, is determined by the stiffness of the implant (i.e., the Young’s modulus multiplied by the geometry of the implant). In this context, a higher strength can reduce the cross-sectional area (or second moment of inertia, depending on the loading type) of the implant to meet the mechanical demands, thereby reducing its stiffness (Figure 1). The strengthening (while maintaining the Young’s modulus) of the material benefits in the same direction as the decrease in the Young’s modulus. Therefore, the strategy of increasing the solid solution strengthening while maintaining the *e*/*a* can work well in the development of bone implant materials. Future research on corrosion resistance and fatigue properties should be conducted and improved to meet the requirements of dental and orthopedic implants.

## 4. Conclusions

In this study, we designed alloys in which Ti is partially replaced by Zr, based on ISO-approved β-titanium alloy Ti-15Mo-5Zr-3Al. The mechanical and biological compatibilities were compared to those of Ti-15Mo-5Zr-3Al and Ti-6Al-4V, which are frequently used as metallic biomaterials. The designed alloys exhibited identical or reduced Young’s moduli and higher strengths, suggesting improved mechanical osteocompatibility. The biocompatibility of the alloys was as good as or better than that of Ti-6Al-4V.

## Figures and Tables

**Table 1:** Atomic radii and valence electron numbers of elements.

Alloy	Ti	Zr	Mo	Al	V
Atomic radius, $r_{i}$ (Å)	1.462	1.603	1.363	1.432	1.316
Valence electron number, *e* (-)	4	4	6	3	5

**Table 2:** Composition and electron–atom ratio (*e*/*a*) of the alloys.

Alloy	Ti (at%)	Zr (at%)	Mo (at%)	Al (at%)	V (at%)	*e*/*a*
Ti-15Mo-5Zr-3Al	83.31	2.84	8.10	5.76	-	4.10
Zr0	86.15	0	8.10	5.76	-	4.10
Zr25	64.61	21.54	8.10	5.76	-	4.10
Zr50	43.07	43.07	8.10	5.76	-	4.10
Zr75	21.54	64.61	8.10	5.76	-	4.10
Ti-6Al-4V	86.20	-	-	10.20	3.60	3.93

**Table 3:** ${∆H}_{mix}$ and δ for each alloy.

Alloy	${∆H}_{mix}$ (kJ/mol)	δ
Ti-15Mo-5Zr-3Al	−7.27	2.55
Zr0	−7.16	1.89
Zr25	−8.00	4.62
Zr50	−8.83	5.45
Zr75	−9.66	5.45
Ti-6Al-4V	−11.03	1.93

## Data Availability

Data will be made available upon request.
